# Internationalisation of the curriculum in health programs

**DOI:** 10.1186/s12909-023-04271-8

**Published:** 2023-04-26

**Authors:** Andrew Keith Davey

**Affiliations:** Griffith Health, Griffith University, G40_8.43, Gold Coast Campus, Southport, QLD 4222 Australia

**Keywords:** Internationalisation, Curriculum, Health, Intercultural Competency

## Abstract

Internationalisation is a broad term that has been used to encompass a range of activities including international student recruitment, student mobility and exchange, international teaching and research collaborations, institutional partnerships, and embedding international and/or intercultural perspectives within curricula.

There are numerous drivers for institutions to develop an internationalisation strategy including building global reputation and influence, having a positive influence on communities, income generation, and helping their students gain a global perspective or develop intercultural competencies. Health students benefit from internationalisation activities as they will enter a workforce that increasingly engages with global diseases and works within multicultural societies.

However, there are risks associated with internationalisation that stem from disjointed institutional decision making, power imbalances, and neo-colonial attitudes. There are also multiple barriers to effectively engaging in internationalisation including individual student circumstances, staff and institutional preparedness, and geopolitical factors.

Within this broader context, internationalisation of the curriculum (IoC) is aimed at incorporating international, intercultural, and global dimensions into the curriculum, including consideration of content, teaching methods, learning outcomes, and how these are supported at a program and institutional level. This is a major undertaking requiring alignment of philosophy between teaching academics, senior university leadership, and the relevant professional body. Examples of IoC within health programs, and the significant challenges involved, are critically discussed in this paper, and strategies to overcome these challenges highlighted.

Whilst recognising the challenges, this paper concludes that undertaking purposeful IoC is a critical step towards ensuring that the future health workforce is adequately prepared for the 21st Century environment.

## Synopsis

There is recognition that practitioners from health and health-related disciplines increasingly work within multicultural environments and/or encounter global health challenges. It follows that universities and other educational institutions need to develop strategies to equip health students with a high degree of cultural competency so that they will be effective within the modern healthcare sector, and have the skills to operate in a global environment. Despite this, internationalisation in health programs is often patchy, overly reliant on enthusiastic individuals, potentially counterproductive, and/or only available to small subsets of students.

To provide context to the opportunities and challenges, the introductory section of this article presents an overview of internationalisation within the education sector. This overview includes a critical discussion of both benefits and barriers to internationalisation, particularly in relation to health. For the purposes of this discussion health includes medicine, nursing, allied health, health sciences and social care.

Internationalisation of the curriculum (IoC), as a subtopic of internationalisation, is then discussed in depth as a mechanism to help provide graduates with the necessary skills and attributes to work within the modern healthcare sector. Examples of IoC from a range of health disciplines are provided and critiqued. The significant challenges relating to IoC are explored with recommendations for, and examples of, success at a program level.

## Introduction

### What is internationalisation?

Internationalisation is a term that, within tertiary education, has been used to encompass a range of activities including international student recruitment, student mobility and exchange, international teaching/research collaborations, institutional partnerships, and embedding international and/or intercultural perspectives within curricula [1–5].

### Why engage in internationalisation?

Institutional drivers to engage in internationalisation activities are varied. They may be values-based, an opportunity to enhance profile and reputation, or financially driven [1]. There are also a range of potential benefits for communities, industry and governments. Below, a number of these key drivers are critically discussed.

International activities present an opportunity to make a positive impact in the world including research on underfunded diseases, helping to improve healthcare practices in developing countries, student engagement with local communities, or education to address local needs. International alumni who return to their home country and move into positions in industry, government, or academia, also represent a conduit for exporting institutional values and creating linkages with overseas institutions. Through such mechanisms there is the opportunity to build global influence. This has potential benefits that flow back to the university including improved student recruitment, access to funding, recognition of professional programs, research collaborations, and institutional global ranking [6–8].

Whilst having high-level goals around global impact and reputation are components of many institutional strategic plans, more often the benefits of internationalisation are considered within the local context [3].

Depending on the country, the income derived from international student fees can be considerable, and where there are limits on domestic places or fees, the international student income cross-subsidies the education of the domestic students and university research productivity [1]. The revenue generated can be high enough to significantly impact a country’s economy. For example, prior to the impact of the Covid-19 pandemic, international education was Australia’s 4th largest export industry with a value of over $37 billon p.a. [9]. Even for countries, such as Japan, that do not traditionally rely on international student income, the potential revenue from international students is increasingly being seen as a way of offsetting declining domestic student enrolments [10].

In addition to the potential income derived from international students, they bring benefits to the classroom by exposing local students and staff to cultural insights and perspectives that they may not have had otherwise [11–13]. This is important in health programs as practitioners need the skills and knowledge to be effective in a culturally diverse and globalised world [11, 14, 15]. However, simply having international students in the class without having a strategy of purposeful engagement is not sufficient to develop the required competencies [16, 17]. The second part of this paper will discuss this in depth.

International students also provide an invaluable workforce for local industry sectors during their study and following graduation. Their importance to the workforce while studying was highlighted during the Covid pandemic, with the Australian government moving away from the cap of 40 h per fortnight for student visa holders to allowing students to work unlimited hours. These moves were primarily aimed at taking pressure off businesses struggling to find workers in sectors such as tourism, retail, agriculture, hospitality and health. The additional work opportunities helped students financially during this period of hardship, but were criticised as contrary to the ethos that students should be focussed on studying high quality programs rather than being engaged as cheap labour and potentially open to exploitation [18].

International students can also play an important role as part of a country’s skilled migration strategy. There are clear advantages to the host country in utilising locally trained international students to fill their skills shortages [1, 19, 20]. In contrast to migrants who are overseas trained, graduating international students have received their tertiary education within the context of their host country; have been subject to the same educational standards and regulatory environment as the local population; are familiar with the nuances of local industry practices; will be eligible to join the relevant professional body; are used to interacting with the local population; and will have developed a network of peers, social support and industry connections [20]. A number of these factors are particularly important for the health professions: students study in nationally accredited programs; there are national differences in legal-ethical operational frameworks; health/disease profiles will reflect the local population and environment; and protocols and equipment vary between different settings. There is far less risk in employing an international student graduating from a local educational institution, who may already have links to the employer, compared to somebody who is overseas trained and is an unknown element. Furthermore, attracting skilled migrants via the international student pathway means that they spend years contributing to the economy via fees and living expenses before formally becoming a migrant.

Following this, having a strategy of attracting high-achieving international students who then convert to skilled migrants is often considered as a win–win, with the student gaining opportunities that may not be present in their home country and the host country accessing talent that will help meet their economic and population growth targets [19]. However, this does open the question of the impact on the home country of losing these high achieving students [3, 21]. Does this brain drain exacerbate existing global inequalities, with wealthy countries continuing to draw young talented individuals from around the world while poorer regions lose their best and brightest? Or do enough internationally trained students return to their home country, bringing fresh ideas, new ways of doing things, and providing a source of skills that their home country does not have the capacity to develop? Such questions raise issues around ethics and equity that need to be considered.

Further equity issues are apparent when considering the prohibitive cost of student fees, flights, accommodation, and general living expenses, particularly in key destination countries such as Australia, UK, Canada, and USA. Only the wealthy can access this education and the advantages it affords, which further widens income disparities. Government, industry or institutional scholarships can help, but government scholarships may come with conditions such as being bonded to the public system post-study, and institutional scholarships impact the economic advantages gained through international student fees.

To offset inequities in access to education, institutions may engage in ventures such as offshore branch campuses, joint degrees, and franchising, where students can study at local prices. This can be desirable where the primary goal of the student is to gain a qualification from an international university rather than to travel, have an overseas experience, or migrate. However, delivering programs offshore presents significant challenges as the institution will be operating in an environment with different education, business and employment laws; unique professional accreditation requirements; and cultural differences in business practices [22]. Ensuring adequate staffing, infrastructure, quality assurance and a reasonable business model is needed to ensure sustainability and prevent reputational damage.

Where offering offshore programs is not an option, articulation programs provide a compromise where the first part of the degree can be obtained relatively inexpensively in the student’s home country but the student will finish the degree as an international student in a destination country of choice. However, there are particular challenges faced by students who move countries part way through a program as they transition into a new academic and cultural environment at a non-typical time point in the student cycle [23]. There is also the question of the value of articulation arrangements for the institution where the students start their education. A typical 1 + 2 or 2 + 2 arrangement means that the local institutions lose 2 years of income from the students who transfer overseas. Therefore, it is important that such arrangements are developed as part of a genuine partnership with mutual benefits rather than as a one-sided recruitment strategy where the institution from the more developed nation is the major beneficiary [3].

In time, inequalities around access to international educational opportunities will be reduced alongside the increasing international reputation of universities outside of the Anglosphere, combined with their much better affordability of fees and living expenses, which is leading to increasing demand for these institutions [24]. Furthermore, the growing number of programs taught in English also increases accessibility and transferability, which helps to increase the options available to international students [10, 25].

Given that the majority of students will complete their degree program in their home country, study abroad experiences, including industry placements, community projects, field trips, and academic programs have been used to develop a global perspective amongst students [26]. In Europe, the ERASMUS scheme has seen millions of students have an international study experience since its inception in 1987 [27]. In Australia, schemes such as the New Columbo Plan provide support for over 10,000 Australian students to engage in projects across the Asia–Pacific in a typical year (https://www.Dfat.Gov.Au/people-to-people/new-colombo-plan/mobility-program/previous-rounds). Similarly, there has been strong growth in study abroad projects in North America and elsewhere in the world [26, 28]. However, such programs have been criticised as being peripheral to teaching programs, only available to limited numbers of students, and a lack of evidence that short term mobility programs lead to transformations in students’ global and intercultural capabilities [29–31]. Nevertheless, study abroad within health programs does appear to be effective if it is faculty led, takes students out of their comfort zone, is immersive, and has reflective practices embedded to facilitate transformative learning [28, 29, 31–33]. From this, there needs to be serious consideration of the underlying pedagogy if such experiences are to have the intended learning outcomes. Interestingly, study-abroad experiences have been linked to enhanced employment outcomes [26, 27]. The implication is that enhanced employability is due to development of intercultural competencies, broadening of the mind, development of a more interesting CV, and greater confidence in interacting with others, but alternatively it could simply be a reflection of the characteristics of the people that seek out study abroad opportunities [27, 34].

### Internationalisation barriers and risks

There are barriers to internationalisation that exist on many levels including government policies, public sentiment, institutional and/or staff resistance, and a multitude of student factors. There are also significant risks to students, institutions and communities that need to be managed [35]. Furthermore, health programs present additional challenges relating to legislation, accreditation, transferability of training, and risks to vulnerable members of the public. Below, a number of these issues are discussed, particularly where they relate to health degrees.

#### Government and political

The economic importance of international students, their key role within migration strategies, and the ability to exert ‘soft power’ through internationalisation activities, means that political decisions can influence international engagement and recruitment.

Sanctions have a major impact on educational participation rates in targeted countries, and this extends to scholarly activities such as the ability to attend conferences, publish research, develop partnerships, as well as access to equipment and educational software/databases [36]. Similarly, as governments seek to counter foreign interference, university international relationships and activities will come under greater scrutiny to ensure that they are within the national interest [37–39].

Students have been used as a political tool when diplomatic relations between counties break down. Examples include the withdrawal of sponsored Saudi Arabian students from Canada following Canadian criticism of Saudi Arabia’s detention of human rights activists [40], and the Chinese government actively dissuading Chinese students from studying in Australia following Australian government calls for investigation into the origins of the Covid outbreak [6, 41]. Conversely, students studying within a country can be used as a mechanism to influence public, academic and political opinion or facilitate transfer of sensitive technologies [6, 37].

Government policy shifts can change the relative attractiveness of a destination country. Post study works rights (PSWR) are an important consideration for students, and changes in PSWR are a major determinant of international student demand for degrees, and the mix of students in different areas of study [21]. Thus, institutions can be impacted by decisions in their own and in competitor countries as changes in PSWR alter the relative attractiveness of destinations. An even greater impact than PSWR on the attractiveness of destination countries was the closing of international borders during the Covid-19 pandemic, with those countries keeping their borders open, or re-opening earlier, being more attractive destinations and recovering market share more quickly than those that remained closed [42].

#### Accreditation

A significant disincentive for students considering studying health programs overseas is that professional bodies may not recognise qualifications or professional licences from other jurisdictions. In some cases, accrediting bodies publish a list of recognised international programs, but more often there is a lack of transparency regarding which foreign qualifications are recognised and the conditions that graduates must meet for them to gain professional registration when they return home. Having examples of alumni who have returned and successfully registered in their country of origin should help increase confidence amongst prospective students, but it is not possible to guarantee that registration rules, or their application, will remain consistent. The situation becomes even more complex when considering establishing joint programs or similar transnational education initiatives, which can trigger additional requirements and costs to ensure recognition, including site visits by the accrediting authorities of each country. However, it is possible to overcome such barriers. Manipal University’s medical program provides an excellent example with students completing the first 2 years of study in India and their clinical training in Malaysia [43, 44]. Most of the students are Malaysian and go on to practice in Malaysia.

A major constraint on internationalisation of health programs is that curricula must adhere to the requirements of accreditation bodies, which can significantly reduce flexibility. A highly prescribed program may not have room for outward mobility experiences; will likely have pre-requisites and course sequencing that does not align well with potential exchange partners, making both inbound and outbound student exchange challenging; and overseas placements may not be recognised by the home accrediting body [1, 11, 26, 34]. As accreditation agencies move towards recognition of learning outcomes rather than insistence on particular content [45], this should open up opportunities for health programs. Furthermore, accreditation standards for health programs increasingly stipulate the need for students to develop intercultural competencies, which will help to contribute towards internationalisation of the curriculum [14, 15, 28, 29, 46–48].

#### Perceptions of International Students

There are a range of cliches that can negatively impact the perception of international students by academics, university staff, domestic students, placement providers, and the broader community, which can result in resistance to internationalisation efforts [20]. These cliches include international students not wanting to integrate, being rote learners, working as taxi drivers rather than studying, having poor language skills, only seeking migration pathways, or not being a good cultural fit with local practices [19–21, 49, 50]. Such stereotypes devalue the multiple complex characteristics and motivations of international students and lead to poor outcomes for all stakeholders [20]. The sector needs to work together to overcome these negative perceptions.

International students are generally seeking to engage [12, 51] but there needs to be the right balance of nationalities in the classroom and mechanisms such as orientation-week, buddy systems, clubs, societies and events that facilitate these interactions [12, 21, 52, 53]. Such mechanisms may also help to mitigate the period of cultural adaptation that students who are new to the environment need to overcome [12, 23, 51–55]. However, it should be recognised that there are differences in the ability of students to adapt depending on the closeness of the culture between their home and the study destination, as well as a range of internal factors and motivations [56].

Poor communication skills are cited by some academics as a reason for not wanting international students. There is no question that appropriate language entry standards need to be set, especially within health degrees. However, academics and preceptors may also benefit from training and support in teaching students from diverse backgrounds [3, 4, 20–22, 49, 54]. For example, a respectful demeanour within a classroom setting could come across as poor communication skills. Certainly, the author has had the experience of engaging with international students who were highly articulate outside of the classroom, only for them to give a very stilted performance in OSCE or viva voce assessments, and then revert to their normal articulate selves once the tape recorder is turned off and they believe that the assessment is over. A teacher with the skills and willingness to make such students feel comfortable and to open up in the classroom will have a very different teaching experience to one who assumes that lack of language skills is what is preventing the student from engaging [12, 22]. Similarly, it has been suggested that the tutor’s personality may be an important factor contributing towards the engagement and learning outcomes of Asian students in Problem Based Learning tutorials [49].

Arguments against having international students in health programs which are based on them having English as a second language or a different accent can also be flipped if consideration is given to the makeup of the local community. Students and graduates who are multilingual should be considered as an asset to a health system that operates in a multicultural society. For example, approximately 30% of the population of Australia were born overseas [57], tourism is a major industry, and there are up to 170,000 ELICOS students who do not have fully developed English language skills [58]. Hence, it is inevitable that health providers will regularly encounter patients who do not have the English capability to adequately describe symptoms, co-morbidities, medical history, etc. Practitioners who can speak to the patient in their own language, and ideally understand some of the cultural nuances that influence the patient, are invaluable in these situations. More work is needed to educate employers, preceptors, academics, and the public of this advantage of training international students to work in the health sector. Moreover, discussions with prospective students, their parents, career counsellors, and recruitment agents, can be useful in highlighting these scenarios and how, with some skills in self-marketing, international students can plan in their career trajectory with confidence rather than feeling that they are at a disadvantage to locals.

#### Student mobility

Student mobility provides opportunities for students to cross borders to undertake part of their study. This can include short term study tours, placements/internships, short courses, and longer term study programs. The immersion in a different culture can bring benefits including knowledge acquisition, cultural insights, and personal growth [26, 29], but there are also inherent risks [28, 35, 59–62]. Risks include cultural imperialism, imposition on the host’s resources, lack of benefit for, or damage to, the host community, culture shock, harassment, sickness, crime, political and industrial events, logistical problems, home sickness, and ethical challenges. Particularly concerning is the potential for health students to be involved in delivering interventions that they are not yet trained to perform, including some instances of surgery by junior medical students [59]. Participants in short term mobility projects can cause significant harm, especially where they move from a highly regulated country to a less developed region. Problems include engaging in activities that they are not qualified or licenced to undertake, culturally insensitivity, or not being respectful of local expertise [61]. Conversely, students moving to a highly regulated environment find that they have limited learning opportunities within the clinical setting due to local restrictions and practices, and often do not receive the same support from host institutions in high income countries as their counterparts receive when being hosted by institutions in low income countries [62].

Some of the risks associated with short term mobility are increased by a lack of appropriate pre-departure preparation, pressure to increase the number of placement opportunities, lack of support while on placement, and/or the absence of post-placement debriefing or reflection [59]. Some issues associated with student mobility can be overcome by online options, particularly given the greater experience with online mobility during the Covid-19 pandemic. However, despite greater acceptance of online student mobility, these activities do not replicate many of the experiences students have during physical internships including interactions with the local populations, patients and their families, and experiencing sights, sounds, smells, and environmental conditions that impact on living conditions and, hence, health outcomes.

#### Student factors

International students are often quite advanced in understanding the value of international experiences for their career and life goals, and are often intentionally seeking out opportunities to engage with students from different cultures [12, 16, 19, 23, 52]. However, for all students it is important that there is a good balance of different nationalities and cultural backgrounds within the class [63]. Large numbers of students from any particular country can be a barrier to the integration and interaction with locals that many international students seek; is considered to be a factor of deteriorating English language capability of international students during the progression of their degree; and may be a cause of resentment by local students who feel that their own educational progress is being impeded [50, 63]. Perceptions, whether real or imagined, that international students receive special dispensations in entry standards or assessment because they pay high fees can cause resentment, as can narratives around international students slowing down the class or needing to be carried in group assignments [16, 50]. In relation to curricula, some students may not see the value of developing a global perspective or associated soft skills, preferring the teaching to default to the foundational knowledge of their profession [34]. However, there does seem to be growing recognition of the importance of a global perspective amongst health students, either as part of core curriculum or as an optional extra, and the potential for this to improve employability [34].

The individual circumstances of the students can also impact their ability to engage in internationalisation activities. Studying oversees for extended periods can be prohibitively expensive for most people [15, 30]. Study tours, short courses, internships, and other mobility options offer opportunities that are more affordable, particularly when sponsorship is available. However, students who are working or caregivers may not be able to take time away. Perceptions of safety, social structure, facilities, and other reputational factors are also important determinants for students considering studying abroad [64].

#### Institutional dynamics

Many of the negative effects of internationalisation can be an unintended consequence of organisational decision making, whereby decisions are implemented without fully understanding how they will address current challenges, impact stakeholders, or create new problems [35]. The ‘garbage can model’ of decision making, characterised by problematic preferences, unclear technology and fluid participation, that is often a trait of tertiary institutions [65], does not align well with the highly nuanced field of international health education which involves multiple stakeholders who may have quite different needs and priorities [35].

Institutional partnerships are often formed between institutions in high income countries and low income countries which sets up the potential for inequitable power dynamics and neo-colonial attitudes [62]. This can, for example, lead to many of the risks and attitudes associated with short term mobility tours whereby the student experience is the primary outcome rather than long term improvements in local community health systems [61]. Furthermore, staff and students participating in the activities can view themselves as superior, and there to help the less fortunate, rather than appreciating that they have much to learn from local expertise and that activities should be mutually beneficial [61, 62, 66]. This “perpetuates a misguided and colonial power dynamic that closes participants’ eyes to host country ways of seeing and doing that could benefit high income countries” [61].

Overcoming counterproductive institutional ways of thinking and acting requires deliberate strategies of deep and genuine partnership engagement where all parties participate in developing shared objectives [35, 62]. This includes strategies to decolonise global health and global health education, ensuring equitable outcomes for all partners [62]. An important aspect of this is designing curriculum to develop greater self awareness and deeper awareness of others, recognise diversity of knowledge, and gain cultural humility [62, 66]. Of particular importance, study tours should follow the principles of guidelines such as the Brocher Declaration which emphasises mutual partnerships; empowering the host country to define the needs and activities; capacity building and creating sustainable programs; compliance with relevant laws and ethical standards; cultural humility and respect; and accountability [61, 67].

### Internationalisation of the curriculum

As discussed above, internationalisation covers a broad range of activities and considerations. Within this broader theme, internationalisation of the curriculum (IoC), and similar initiatives such as internationalisation at home, are aimed at incorporating international, intercultural and global dimensions into the curriculum, including consideration of content, teaching methods, and learning outcomes, and how these are supported at a program and institutional level [17, 68].

The term IoC can be interpreted in different ways [68]. Whereas internationalisation at home suggests that study abroad or exchange are not included within this term, IoC can be inclusive of such activities. There is variation within the literature regarding whether IoC is mainly aimed at international students or whether it is intended for the general student population. However, the concept that IoC should be primarily aimed at international students is generally considered to be too narrow, and misses opportunities for developing intercultural competencies in the domestic context [69]. All students, whether domestic or international, need the skills and attributes to operate effectively in a globalised world, including within multicultural societies. Incorporation of insights from international or culturally diverse students into the classroom can be powerful, but having international students in the class should not be a prerequisite for IoC [69]. Indeed, the definition of an international student is somewhat arbitrary, often based on visa status or the type of fees that the students are paying rather than on their cultural background or life journey [3, 70]. International and domestic students can both be highly heterogenous groups, and this needs to be recognised when designing curricula and within the broader university context [70].

If all graduates need attributes such as having a global perspective and intercultural competency to be successful in their career, IoC should be part of core courses and the skills developed throughout the degree, rather than being consigned to electives or specialised options for a few students [68, 69]. A deliberate strategy of incorporating intercultural competency training into health programs develops insights into areas such as the role of culture on practitioner-patient interactions, cross-cultural communication styles, the challenges facing new migrants when accessing healthcare systems, and an appreciation of the level of cultural diversity within local populations [14, 46–48]. One example of this is described by Haines et al. who discuss the use of videos which explore cultural attitudes to dying with terminal illness in Morocco and the Netherlands and how this influences communication by the doctors in those countries [71]. Such insights often increase empathy towards others, reduce anxiety when interacting with people from different cultures, and better equip students for practicing as health professionals in a multicultural society [29, 46, 47, 54].

Having internationalisation embedded within core curricula does not prevent interested students gaining extension to this core training, including through mobility opportunities. Arguably they will be better prepared to benefit from these mobility experiences if they have early exposure to internationalisation in their degree, and could even be a useful resource to feed back into the core curriculum on their return from their overseas experience. Hence, “mobility needs to be seen as adding value to an internationalised curriculum, not as the focal point of internationalisation efforts” [72].

### Embedding IoC in health programs

Some IoC could potentially happen organically through having international students in the classroom, academics with international backgrounds or experiences, interaction with visiting scholars, or initiatives introduced by enthusiastic individuals [11, 34, 73]. For example, an occupational therapy class on care of the elderly may create incidental discussion on the place of the elderly in different cultures, including attitudes towards family-based vs institutional care (personal communication). Organic relationships can provide a strong foundation for development if there are systems in place to help build and sustain them, but relying exclusively on organic growth will lead to patchiness in the application of internationalisation within curricula, tends to be inefficient as different areas ‘re-invent the wheel’, may not necessarily result in best practice if ideas and experiences are not shared, and will have sustainability issues if over-reliant on an individual staff member and/or are not adequately resourced. Ad-hoc initiatives and interactions to internationalise the curriculum may be better than having no internationalisation activities, but is not a long-term option if we are serious about developing high quality and sustainable activities [5, 73]. Successfully embedding internationalisation within degrees requires staff buy-in to develop a broad and consistent application, and institutional backing to ensure quality and sustainability [5, 17, 46, 74].

#### Staff buy-in

There can be different perceptions of the value of IoC depending on the academic discipline [4, 21, 22]. Furthermore, it is easy for academics to put up reasons for not introducing elements of internationalisation into programs including: accreditation requirements; pushback from preceptors or WIL placement sites; overfull curricula; excessive workload; or a lack of relevance to their particular discipline [4]. Some of this resistance may be underpinned by parochial attitudes of staff, which has been identified as a major barrier to IoC [5, 51]. A shift in culture whereby staff understand, value and embrace internationalisation, can see such arguments dissipate, particularly where the degree program is looked at holistically [22]. Developing such a culture is helped by having staff who have an international background, speak multiple languages, who have been active in international programs, and see the value of internationalisation to the student rather than thinking purely from a disciplinary context [4]. However, engagement in strategic and operational planning to develop a sense of ownership of the internationalisation agenda will likely be required to develop a common purpose and overcome resistance [5, 11, 17, 68]. Without this level of buy-in, any top-down initiatives, including linking to graduate attributes or accreditation requirements, have the potential to default to a tick-box exercise [17, 75].

#### Institutional backing

It is increasingly common for universities to have references to ‘global’ or ‘international’ within their vision and mission statements and as part of their graduate attributes [17]. However, institutional mission statements are often not backed by practical support [4, 5, 30, 68, 73]. If universities see the value of internationalisation then there needs to be investment of resources and incentives to encourage and reward staff [76]. This includes having internationalisation-related work recognised in areas such as workload allocations, departmental KPIs, performance management reviews, staff training, opportunities to gain experience, promotion and career progression criteria [4, 51, 76]. This must be underpinned by enabling systems and processes [5, 17], which can require a significant financial investment. Importantly, there needs to be recognition that internationalisation is a long-term investment [76, 77], and that outcomes such as global influence, enhanced international profile, better employability of graduates, and improved patient care within multicultural communities may take years, or even decades, to be realised. This can be a challenge for institutions that are used to measuring success on an annual basis with simplistic metrics such as the number of publications, grant income, students recruited, and student survey scores. It is, therefore, critical that internationalisation becomes part of the fabric of the institution, rather than a sideline activity or viewed as a series of budget lines, if the long-term benefits are to be realised [5, 48, 74].

#### Examples of internationalisation of the curriculum in health

Examples of IoC within health programs demonstrate the potential for improved learning outcomes. However, such initiatives are often limited in scope or impact:

- Mak demonstrated excellent learning outcomes in their Health Psychology course, particularly in relation to enhanced cultural capability, but the course was an elective and undertaken by just 19 students [46].
- Hyett et al.’s virtual learning activities with oral health and occupational therapy students from Australia and Hong Kong involved over 200 students and provided some valuable insights for students in both countries [47]. However, although the respective courses were core material, the interaction was limited to just one 90 min virtual meeting, and it is perhaps not surprising that there was no statistically significant improvement in cultural competency scores.
- A similar concept was utilised in a collaboration between the University of Kentucky and Peking University Third Hospital involving multiple virtual interactions of pharmacy students from the two institutions to compare and contrast healthcare systems, and led to development of skills in overcoming communication barriers [15].
- The concept of compare and contrast as a learning tool was also utilised at Griffith University (GU) where pharmacy students benefitted from a visiting Canadian scholar who enabled the transformation of the Pharmacy Law and Ethics course to include a comparative analysis of Canadian and Australian pharmacy practice [78]. Unfortunately, the return of the visiting scholar to Canada coinciding with the imminent retirement of the primary course convenor, meant that this initiative was not sustained in subsequent years (personal insight). This highlights the inherent risks of reliance on individual initiatives rather than having a programmatic approach.
- Das successfully reconfigured a Language and Communication in Physiotherapy course to move away from a deficit-remediation approach to a more positive aim of building intercultural competency [54]. However, the course was only taken by incoming international students who were all from the Indian subcontinent. While these students seemed to benefit, it does raise the question of whether such examples can be considered as IoC if there is no apparent engagement or learning outcomes for local students. Nevertheless, there are full degree programs that have been developed specifically to recruit international students where the aim of embedding cultural competency is to facilitate the transition of the international student to the local context [21].

These examples, while having their limitations, provide some proof of concept. However, they also re-enforce the notion that a programmatic approach is likely to be more successful in achieving desirable and sustainable learning outcomes than individual academics working in isolation [14, 17]. On the face of it, some programs lend themselves more readily than others to IoC, and this may be compounded by disciplinary ways of thinking [17, 22, 74]. For example, at GU, the name and stated aims of their Master of Global Public Health with its *“strong focus on global health…. electives in areas such as public health nutrition, health promotion, climate change, international health, environmental health, and health services management”* clearly indicates an intention to deliver an internationalised curriculum [79]. Indeed, having a global perspective is not unusual for public health programs [12, 13]. However, for the Bachelor of Biomedical Science which also sits within the GU Health Group, the link to an internationalised curriculum is far less apparent when reading the program overview: *“genetics, biochemistry, cell biology, anatomy, physiology, immunology, microbiology, infectious disease, pharmacology and neuroscience *[80]*.”* Yet, graduates from both programs will enter a globalised work environment and will all benefit from an international perspective and enhanced cultural competency. This is not to say that biomedical science cannot offer an internationalised curriculum. Indeed, all GU students are expected to develop specific graduate attributes during their program including “Graduate Attribute 6: Effective in culturally diverse and international environments,” [81] and one of the first year Biomedical Science courses is ‘Health Challenges for the 21^st^ Century’ which includes aspects of global health. Within the broader biomedical science program there are a number of areas that can be readily adapted to incorporate an international or cultural aspect. For example, pharmacology is influenced by a range of factors including genetics, diet, and lifestyle, and there are insights to be gained from international comparisons of pharmacological interventions [82]; infectious diseases impact different populations in different ways depending on climate, geography, living conditions, and local practices in antibiotic prescribing or vaccinations; and there are different regulatory and operational frameworks for working with chemicals and biological materials around the world. These examples provide opportunities to open discussion that is much broader than the fundamental principles of the subject. The incorporation of broadening subjects, majors or electives, including from the social sciences, also provides opportunities to equip students with the necessary skills to succeed in global industries [77]. It would, however, be helpful if such programs were more explicit regarding the intention to develop intercultural and international perspectives, rather than simply list biomedical science topics [17].

It follows that the initial stages of internationalising the curriculum at a program level should be an assessment of what internationalisation activities currently exist within the program and how they relate to a shared vision of what is meant by internationalisation i.e. student mobility and/or understanding the determinants of global health and/or intercultural competency. Ideally, the definition of ‘internationalisation’ should be as broad as possible and consider not just content but also learning and teaching approaches, with approaches that lead to transformative learning being adopted [14, 17]. There then needs to be a process of development, implementation, and evaluation of curriculum. One example is the framework for change suggested by Leask (2013): Review and Reflect → Imagine → Revise and Plan → Act → Evaluate → Review and Reflect → ….

There are several key principles that are important when developing and implementing the internationalised curriculum. As discussed above, academic buy-in is critical and the changes should be led by the academic team with external input and support as appropriate [17]. Staff should have a good understanding of what IoC actually means and training and support to help them implement change [5, 68]. Initiatives that take a student-centred approach to develop critical and reflective practice in an interconnected and complex global environment should be prioritised [14, 59]. Internationalisation must be embedded in core curriculum and reinforced throughout the program to ensure that every student can develop the required competencies [14, 17, 83]. Electives, study abroad, exchange and mobility programs can then be used to compliment and extend the core curriculum for those students who have the interest and capacity to undertake these activities. However, we need to remain cognisant of some of the ethical grey areas, particularly in the case of outreach activities where power/economic imbalance may exist or the motivations of students may be self-serving and neglect the needs of the community [3, 14, 59]. Similarly, it is essential to avoid stereotypes or to inadvertantly marginalise international or culturally diverse students within the class [4, 14, 29], including racialising disease [84]. Importantly, the impact on learning outcomes of any changes to curriculum should be assessed [5].

From this, it is apparent that IoC at a program-level within health degrees is a major undertaking. Considerations include:

- staff buy-in and upskilling,
- consistent and well understood philosophical frameworks,
- alignment of curriculum, teaching, and assessment with institutional, professional, disciplinary, and interdisciplinary priorities,
- investment in long term support and sustainability,
- mechanisms to overcome the multiple challenges that will arise.

Despite the size of the challenge, different health disciplines have successfully undertaken this exercise. Some examples are provided below.

Many of the elements discussed above were in place for the development of the Bachelor of International Medicine Program at the University of Groningen [71]. There was an imperative from the National Framework on Medical Education to ensure that medical graduates understood the impact of factors such as cultural and ethnic diversity on medical treatment; a strong and longstanding institutional drive to internationalise, along with the realisation that future doctors need to work in a globalised environment in relation to diseases, patients and health workers; a group of highly enthusiastic staff who brought different skillsets; and a culturally diverse student group. The program was based on the existing medical program, providing a solid foundation for development. The teaching team were able to incorporate global health themes throughout the program through problem-based learning approaches and by utilising the student diversity in the classroom. However, a lack of cultural diversity of the academics was a limitation.

Similarly, the School of Nursing at the University of Northern Arizona had a history of international engagement; strong institutional drivers; leadership who provided funding and support; a process designed to bring staff onboard; a clear philosophy that every student should develop the skills to work in a globalised world; and an opportunity presented by a planned curriculum review due to revised accreditation standards [11]. They developed a global health theme that spanned the three levels of the core program. This was supplemented by co-curricular activities and optional international mobility opportunities [11].

Internationalisation of the curriculum is often thought of in relation to traditional degree programs where students come together at a campus [13]. However, online technologies allow interactions of students who remain in their home countries, including in part time programs, short courses, or micro-credentials. This is particularly useful for healthcare workers who are looking to upskill without the need to travel. Gemmell et al. (2015) successfully engaged an internationally diverse group of students in their online public health program in a manner that enhanced the learning outcomes of all students. The success was attributed to clever design of teaching and assessment that encouraged students to interact with students from other countries.

## Conclusion

There is growing recognition that health professionals need to operate in a global environment and require a high degree of cultural competency to be effective within the modern healthcare sector. Despite this, IoC in health programs is often patchy, overly reliant on enthusiastic individuals, and/or only available to small subsets of students. To truly internationalise the curriculum is a major undertaking and there are multiple barriers that need to be overcome. Where there is alignment of philosophy between academics, senior leadership, and the relevant professional body, it is possible to embed internationalisation themes throughout health programs. This process is helped by having a systematic approach, a history of international engagement, staff with an international perspective, and a degree of pragmatism such as utilising planned curriculum reviews to avoid workload duplication. It is apparent that IoC requires learning and teaching approaches that are transformative, and so consideration of teaching and assessment modalities is as important as the content. Furthermore, learning outcomes and their impact on professional practice need to be assessed and inform further development of the curriculum. These challenges must be met if we are to adequately prepare our students for their future, ensure that the health workforce is equipped for the 21^st^ Century, and for institutions to capitalise on the long-term benefits that internationalisation brings.

## Data Availability

All materials are publicly available online.
